# *ITIH5* and *ECRG4* DNA Methylation Biomarker Test (EI-BLA) for Urine-Based Non-Invasive Detection of Bladder Cancer

**DOI:** 10.3390/ijms21031117

**Published:** 2020-02-07

**Authors:** Michael Rose, Sarah Bringezu, Laura Godfrey, David Fiedler, Nadine T. Gaisa, Maximilian Koch, Christian Bach, Susanne Füssel, Alexander Herr, Doreen Hübner, Jörg Ellinger, David Pfister, Ruth Knüchel, Manfred P. Wirth, Manja Böhme, Edgar Dahl

**Affiliations:** 1 Institute of Pathology, RWTH Aachen University, 52074 Aachen, Germany; bringezu.sarah@gmail.com (S.B.); laura.dierichs@rwth-aachen.de (L.G.); david@fiedler.online (D.F.); ngaisa@ukaachen.de (N.T.G.); maximilian.koch@uk-koeln.de (M.K.); rknuechel-clarke@ukaachen.de (R.K.); 2 RWTH Centralized Biomaterial Bank (RWTH cBMB), Medical Faculty, RWTH Aachen University, 52074 Aachen, Germany; 3 Department of Urology, RWTH Aachen University, 52074 Aachen, Germany; chbach@ukaachen.de (C.B.); david.pfister@uk-koeln.de (D.P.); 4 Department of Urology, University Hospital Carl Gustav Carus, Technische Universität Dresden, 01307 Dresden, Germany; Susanne.Fuessel@uniklinikum-dresden.de (S.F.); Huebner-Doreen@gmx.de (D.H.); Manfred.Wirth@uniklinikum-dresden.de (M.P.W.); 5 Biotype GmbH, 01109 Dresden, Germany; alx.herr@gmail.com (A.H.); m.boehme@biotype.de (M.B.); 6 Department of Urology, University Hospital Bonn, 53105 Bonn, Germany; joerg.ellinger@ukb.uni-bonn.de; 7 Department of Urology, Uro-Oncology, Robot Assisted and Reconstructive Urologic Surgery, University Hospital Cologne, 50937 Cologne, Germany

**Keywords:** bladder cancer detection, urinary biomarkers, DNA methylation, ECRG4, ITIH5

## Abstract

Bladder cancer is one of the more common malignancies in humans and the most expensive tumor for treating in the Unites States (US) and Europe due to the need for lifelong surveillance. Non-invasive tests approved by the FDA have not been widely adopted in routine diagnosis so far. Therefore, we aimed to characterize the two putative tumor suppressor genes *ECRG4* and *ITIH5* as novel urinary DNA methylation biomarkers that are suitable for non-invasive detection of bladder cancer. While assessing the analytical performance, a spiking experiment was performed by determining the limit of RT112 tumor cell detection (range: 100–10,000 cells) in the urine of healthy donors in dependency of the processing protocols of the RWTH cBMB. Clinically, urine sediments of 474 patients were analyzed by using quantitative methylation-specific PCR (qMSP) and Methylation Sensitive Restriction Enzyme (MSRE) qPCR techniques. Overall, *ECRG4*-*ITIH5* showed a sensitivity of 64% to 70% with a specificity ranging between 80% and 92%, i.e., discriminating healthy, benign lesions, and/or inflammatory diseases from bladder tumors. When comparing single biomarkers, *ECRG4* achieved a sensitivity of 73%, which was increased by combination with the known biomarker candidate *NID2* up to 76% at a specificity of 97%. Hence, *ITIH5* and, in particular, *ECRG4* might be promising candidates for further optimizing current bladder cancer biomarker panels and platforms.

## 1. Introduction

Bladder cancer is the most frequent urogenital malignant tumor concerning both sexes worldwide, with an estimated ~549,400 new cases and 200,000 deaths in 2018 [1], which causes the highest costs of all cancers per patient [2]. In the European Union (EU) alone, the costs were €4.9 billion, with health care accounting for €2.9 billion in 2012 [3] due to long-term survival with the need for lifelong surveillance by cost-intensive diagnostically tools [2]. Cystoscopy, the “gold standard” for the detection of bladder cancer, is an invasive and time-consuming procedure, achieving an operator-dependent sensitivity and specificity of approximately 90% [4]. In particular, repeating cystoscopy for patients with non-muscle invasive bladder cancer (NMIBC) to determine whether their disease has recurred or progressed to muscle invasive bladder cancer (MIBC) represents a major cost associated with treating bladder cancer patients [5]. Nevertheless, only 10% of haematuria patients are faced with a diagnosis of bladder cancer [6], a fact that did not increase the compliance of undergoing cystoscopy. Complementary to these procedures, the current guidelines recommend completion by non-invasive urine cytology, which, however, is characterized by poor sensitivity varying between 20 to 53% [7]. Additional non-invasive urinary assays have been developed, which could help to minimize the invasive procedure of cystoscopy and reduce its economic burden (for an overview see: [8]). Although such assays have been shown to increase the sensitivity of urine cytology, they have not been widely adopted in routine practice: either they are characterized by cost-intensive performances, like UroVysion [9], or failed as point of-care tests due to limited sensitivity or specificity, such as the NMP22-based “BladderCheck^TM^ Test” [10,11]. Given that, none of the currently available urinary biomarkers that have been approved by the FDA can absolutely be recommended as a stand-alone test to replace cystoscopy in the clinic. Recently, several commercially available tests have been developed with improved sensitivity and specificity by using mRNA (e.g., “Xpert BC” [12]) or protein-based ELISA assay technology (e.g., “UBC” [13]), but these data must be independently be confirmed in further studies. Therefore, it is still of great interest to identify novel tumor biomarkers for urine-based early detection of bladder cancer, which might optimize existing panels and platforms to improve both the initial detection of bladder cancer and detection of its recurrence.

For several decades now, epigenetic alterations are an excellent source of biomarker candidates for cancer detection, diagnosis, and prognosis [14]. In particular, aberrant DNA hypermethylation of putative tumor suppressor genes emerged as a potential biomarker source for assessing early cancer detection, which has recently moved towards clinical practice, for instance, in colorectal cancer [15]. For non-invasive detection of bladder cancer, promising DNA methylation biomarkers have been described in various studies [16], but the FDA has approved none of the presented methylation biomarkers (panels) for routinely diagnostic procedures so far. In the presented study, we focused on two putative tumor suppressor genes in bladder cancer that may also hold a prognostic impact, namely inter-α-trypsin inhibitor heavy chain 5 (*ITIH5*) and esophageal cancer-related gene 4 (*ECRG4* or *C2orf40*). *ITIH5* has previously been shown to be epigenetically silenced in various cancer entities [17,18,19], including bladder cancer [20], where its expression was associated with tumor recurrence of the clinical important group of high-grade pT1 patients. In addition, *ITIH5* was characterized as a putative metastasis suppressor gene in breast [21,22] and pancreatic cancers [23]. *ECRG4* has also been described to be a candidate tumor suppressor gene that is inactivated by DNA methylation in cancers, like esophageal squamous cell carcinoma [24,25], breast cancer [26], renal cell cancer [27], and colorectal cancer [28,29], but not in bladder cancer so far. 

We now provide evidence that *ECRG4* and *ITIH5* DNA methylation could be useful as urinary biomarkers for non-invasive bladder cancer detection. Biomarkers were assessed by comparing different techniques, i.e., bisulfite-pyrosequencing, qMSP, and MSRE qPCR in comprehensive cohorts of patients with bladder diseases and controls, overall composing 474 urine samples, including a significant number of benign and inflammatory diseases. In particular, we demonstrate strong biomarker performance for *ECRG4*, which might be a suitable candidate to complete and improve current non-invasive biomarker panels and platforms.

## 2. Results

### 2.1. Analytical Performance of ITIH5 and ECRG4 qMSP and Pyrosequencing Biomarker Assays 

*ITIH5* and *ECRG4* have been previously identified as putative class II tumor suppressor genes, which are epigenetically silenced in various tumor entities. In the presented study, we aimed to assess the biomarker quality of both candidates to detect bladder tumors *via* urine samples. The analytical performance of quantitative methylation-specific PCR (qMSP) and pyrosequencing assays was assessed involving standard biobank processing procedure of the RWTH cBMB biobank prior to assessing the biomarker performance of ECRG4 and ITIH5 by patient materials. 

**Figure 1:**
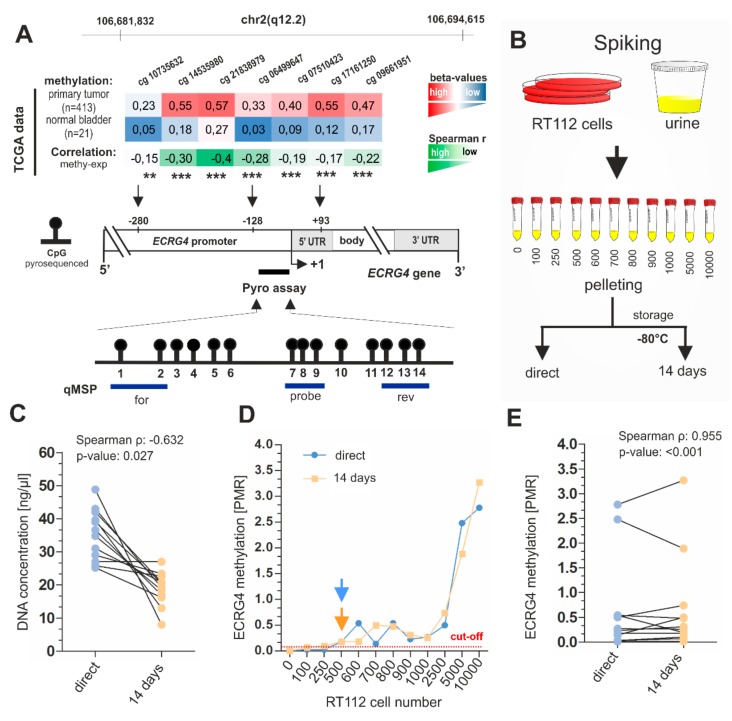
Analytical performance of the Quantitative Methylation-Specific PCR (qMSP) *ECRG4* DNA methylation biomarker assay. (**A**) Schematic map of the human *ECRG4* gene, including the relative positions and median *β*-values (of normal and tumor samples) of seven CpG sites based on 450K methylation arrays of the bladder cancer (BLCA) TCGA data set and corresponding Spearman correlation between *ECRG4* methylation (colored scale bar red to white; red: high methylation, white: low methylation) and *ECRG4* mRNA expression (colored scale bar green to white; green: strong correlation, white: no correlation) for each CpG site; ** *p* ≤ 0.01, *** *p* ≤ 0.001. +1: *ECRG4* transcription start site (TSS). Relative position of the promoter area analyzed by bisulfite-pyrosequencing that comprises 14 single CpG sites (black dots) is shown as a black line. CpG sites analyzed by qMSP (blue lines) were indicated covering the pyrosequenced promoter region close to the TSS. (**B**) Cartoon illustrating the spiking experiment using cultured RT112 tumor cells and urine pooled from four healthy donors. Distinct cell numbers of RT112 tumor cells (100-10,000) were spiked into 20 mL urine, respectively. Afterwards, urine samples were processed according to the standard operating protocol of the RWTH cBMB biobank. Pellets of probe set A were directly used for DNA extraction while urine sediments of probe set B were stored according to the RWTH cBMB conditions (−80 °C) for two weeks before further processing. (**C**) Comparison of DNA concentrations (=yield) achieved of samples from probe set A (direct processing) and B (after 14 days). DNA yield was significantly higher in freshly processed urine samples. (**D**) *ECRG4* promoter methylation determined by using qMSP of spiked urines samples. Red dotted line: threshold value for positive detection; orange and blue arrow: stably exceeding the threshold (detection limit) (**E**) Correlation of *ECRG4* DNA methylation for spiked urine samples of probe set A and B.

For *ECRG4*, Figure 1A shows the genomic location of the qMSP and pyrosequencing assays. Both of the assays spanned CpG sites, which were characterized by strong median hypermethylation in bladder tumors within the TCGA data set [30] as compared to normal controls. CpG sites of the *ECRG4* target region showed a significant inverse correlation between DNA methylation and *ECRG4* mRNA expression (Figure 1A). Subsequently, a spiking experiment was performed to assess both sensitivity and reproducibility in dependence on a distinct number of RT112 tumor cells (range: 100–10,000 cells), i.e., RT112 bladder cancer cells harboring methylated *ECRG4* (see Supplementary Figure S1) and *ITIH5* [20] genes were spiked into 20 mL pooled urine of healthy donors (*n* = 4), respectively. Urine samples that were spiked with RT112 cells were processed according to the standard operating protocol of the RWTH cBMB. Urine pellets were either directly used for DNA extraction (probe set A) or urine sediments were stored according to the RWTH cBMB conditions (−80 °C) for two weeks (probe set B) (Figure 1B). We found that DNA yield was significantly higher in freshly processed samples when compared to those processed after two weeks of storage at −80 °C (probe set B, Figure 1C). However, this was not associated with significant changes in the detection sensitivity of *ECRG4* methylation by both assays, qMSP and pyrosequencing). Overall, the *ECRG4* qMSP assay showed the highest sensitivity with a detection limit of 25 tumor cells/ml (equivalent to 89.75 pg tumor DNA), whereas pyrosequencing-based detection of *ECRG4* methylation required 45 tumor cells/ml (161.55 pg tumor DNA) urine (Figure 1D). Furthermore, the *ECRG4* qMSP assay exhibited a robust reproducibility when comparing methylation detection rates of the two storage time points, i.e., the qMSP assay convinced with high sensitivity and strong reliability (Spearman *r*: 0.955, *p* < 0.001) (Figure 1E). In contrast, pyrosequencing missed a significant correlation of *ECRG4* methylation levels of spiking samples, which were directly used for DNA extraction and those processed after two weeks (Supplementary Figure S2).

**Figure 2:**
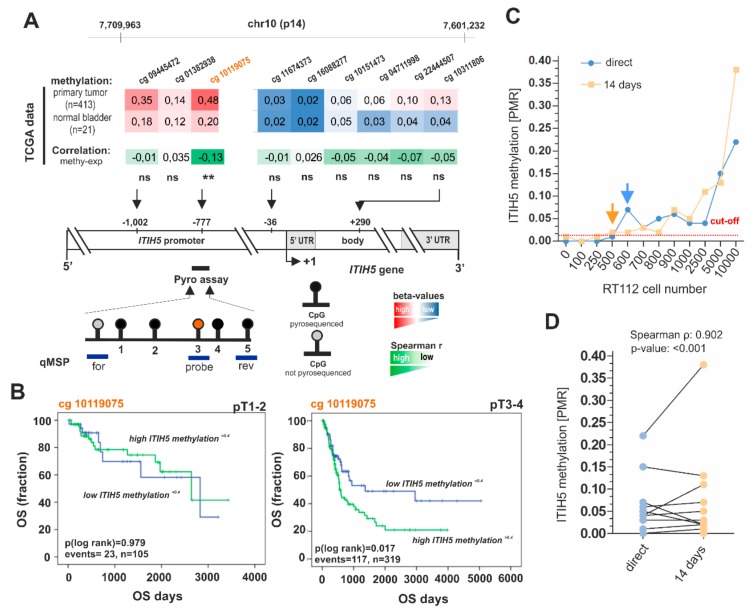
Analytical performance of the qMSP *ITIH5* DNA methylation biomarker assay. (**A**) Schematic map of the human *ITIH5 gene* including the relative positions and median median *β*-values (of normal and tumor samples) of nine CpG sites based on 450K methylation arrays of the BLCA TCGA data set and corresponding Spearman correlation between *ITIH5* methylation (colored scale bar red to white; red: high methylation, white: low methylation) and *ITIH5* mRNA expression (colored scale bar green to white; green: strong correlation, white: no correlation) for each CpG site; ** *p* ≤ 0.01. +1: *ITIH5* transcription start site (TSS). Relative position of the upstream promoter area analyzed by bisulfite-pyrosequencing that comprises five single CpG sites (black dots) is shown as a black line. CpG sites analyzed by qMSP (blue lines) were indicated largely covering the pyrosequenced promoter region. (**B**) Kaplan–Meier survival curves display overall survival (OS) of patients with high ITIH5 methylation (*β*-value of CG #10119075 > 0.4, green curve) compared to low ITIH5 methylation (*β*-value of CG #10119075 ≤ 0.4, blue curve) based on TCGA data. (**C**) *ITIH5* promoter methylation determined by using qMSP of spiked urine samples. Red dotted line: threshold value for positive detection; orange and blue arrow: stably exceeding the threshold (detection limit) (**D**) Correlation of *ITIH5* DNA methylation for spiked urine samples of probe set A and B.

qMSP and pyrosequencing assays for the detection of the *ITIH5* promoter methylation were established similar to *ECRG4*. In Figure 2A, the relative location of qMSP and pyrosequencing primers are indicated. TCGA BLCA data analyses confirmed differences in median DNA methylation level for the targeted region of the *ITIH5* qMSP and pyrosequencing assay, which spanned the CpG site (#10119075), showing an inverse correlation between *ITIH5* DNA methylation and corresponding gene expression (Figure 2A). Of clinical significance, *ITIH5* hypermethylation of the CG site #10119075 was associated with shorter overall survival in advanced (pT > 2) bladder tumors (Figure 2B). In concordance with the results that were observed for *ECRG4*, pyrosequencing-based detection of *ITIH5* methylation failed to perform with a suitable reproducibility when using samples of both storage time points (directly processed vs. two weeks biobank storage). The *ITIH5* qMSP assay achieved strong reliability (Spearman correlation: 0.902, *p* < 0.001, Figure 2C,D) and high sensitivity being characterized by a detection limit that ranged between 25 and 30 tumor cells/mL (89.75 pg/107.70 pg tumor DNA), whereas a robust detection of *ITIH5* methylation by pyrosequencing required at least 125 tumor cells/mL (448.75 pg tumor DNA) urine (Supplementary Figure S2). Hence, qMSP assays of both genes were selected for assessment in a clinical cohort setting.

### 2.2. Clinical Biomarker Performance of the ECRG4-ITIH5 qMSP Test for Accurate Non-Invasive Detection of Bladder Cancer 

**Figure 3:**
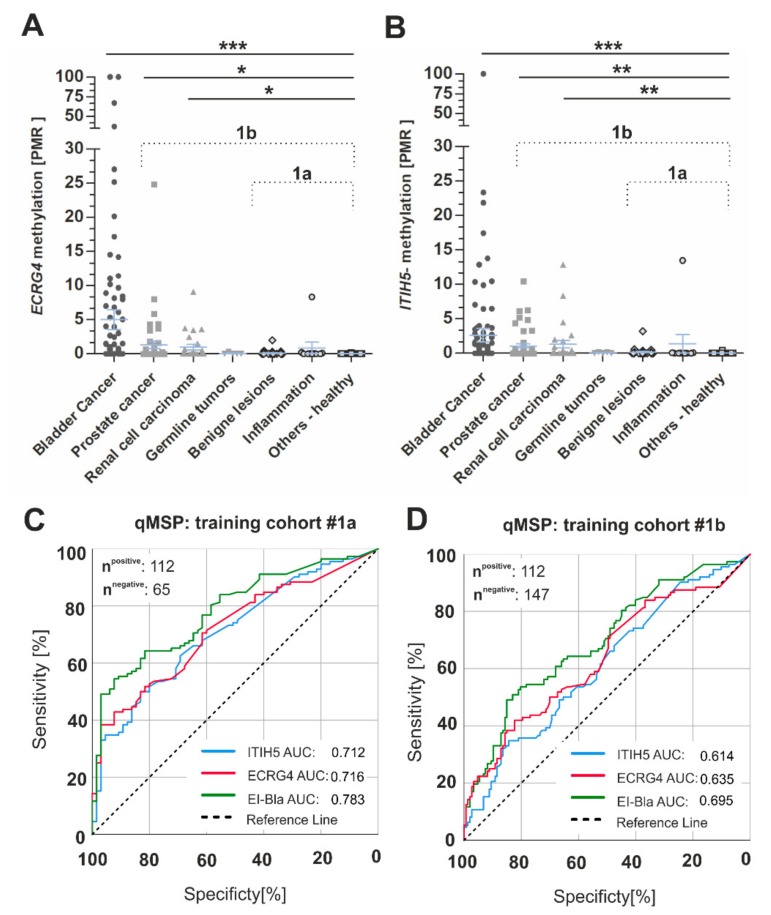
Performance of the *ECRG4* and *ITIH5* biomarker panel using qMSP technique and training cohort #1. (**A**,**B**) Scatterplots show the PMR methylation values for *ECRG4* (**A**) and *ITIH5* (**B**) in urine sediments of urological tumors, benign lesions, inflammatory diseases and healthy samples; *** *p* < 0.001, ** *p* < 0.01, * *p* < 0.05. 1a: training cohort excluding other urological malignancies 1b: training cohort including other urological malignancies. (**C**,**D**) ROC-curve analysis illustrating *ECRG4* (red curve), *ITIH5* (blue curve) and *ECRG4*-*ITIH5* (green curve) biomarker performance based on qMSP in cohort 1a (being and inflammatory controls (**C**)) and cohort 1b (further urological cancer entities as controls (**D**)), *AUC*: Area under the curve.

*ECRG4* and *ITIH5* performance was initially assessed in a clinical cohort of urine samples (total *n* = 263) comprising 116 urine samples that were derived from bladder cancer patients. Patients with urological malignancies of other origin (testis, prostate, kidney) as well as benign and inflammatory urological-diseases (prostate hyperplasia, renal stones, chronic cystitis) and healthy (without pathological finding) donors that were included as controls (cohort 1a: benign - inflammatory, cohort 1b: all controls including urological cancers). *ECRG4* and *ITIH5* methylation for each urine sample is shown as the mean percentage of methylated reference (PMR) in the scatter plots of Figure 3A,B. *ECRG4* and *ITIH5* methylation were both significantly increased in the urine samples from patients with cancers of the bladder (*ECRG4* mean PMR: 5.011, 95% CI: 2.118–7.905; *ITIH5* mean PMR: 2.634, 95% CI: 0.730–4.539), the prostate (*ECRG4* mean PMR: 1.306, 95% CI: 0.178–2.434; *ITIH5* mean PMR: 1.018, 95% CI: 0.441–1.720), and the kidney (*ECRG4* mean PMR: 0.985, 95% CI: 0.188–1.781, *ITIH5* mean PMR: 1.311, 95% CI: 0.147–2.475) when compared to healthy controls (*ECRG4* mean PMR: 0.017, 95% CI: <0.001–0.033, *ITIH5* mean PMR: 0.031, 95% CI: 0.009–0.070). In benign and inflammatory diseases, a single statistical outlier was detected, respectively, however, diagnosis had been done in a clinical setting and, thus, a true malignancy in this few cases cannot be completely excluded. No associations were found between both *ECRG4* and *ITIH5* promoter methylation and clinical-pathological characteristics, including tumor size, histological grade, age at diagnosis, and gender (Supplementary Table S1 and S2). Calculating the optimal cut-off value for a combined *ECRG4-ITIH5* (EI-BLA) qMSP application with robust specificity by using ROC statistics (Table 1), we demonstrated significant discrimination of bladder cancer patients from non-malignant controls (control cohort 1a) with a sensitivity of 64.3% and a specificity of 81.5% (AUC: 0.783, 95% CI: 0.716–0.850, *p* < 0.0001) (Figure 3C). 

Additionally, *ECRG4-ITIH5* in combination were able to distinguish bladder cancer patients from patients with neoplasms of other urological origin (control cohort 1b) with similar specificity (81.6%, AUC: 0.695, 95% CI: 0.631–0.760, *p* < 0.0001) but reduced sensitivity (50.9%) (Figure 3D). The application of both biomarker candidates in combination achieved the most reliable results as compared with single biomarker performances.

**Figure 4:**
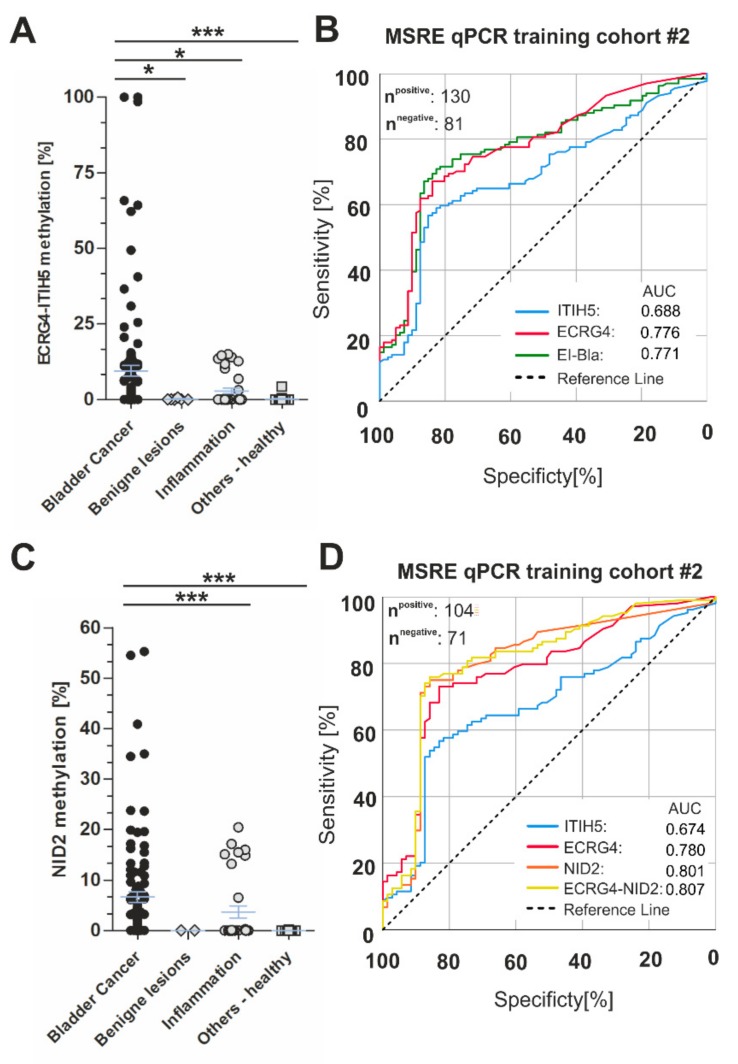
Biomarker performance of *ECRG4*, *ITIH5*, and the *ECRG4*-*ITIH5* panel assessed by an independent urine cohort (training cohort #2) and compared to *NID2* using MSRE qPCR. (**A**) Scatter plot illustrates significant increased methylation levels for *ECRG4* and *ITIH5* in urine sediments of bladder cancer compared to benign lesions, inflammatory and healthy samples; *** *p* < 0.001, *p < 0.05. (**B**) ROC-curve analysis illustrating *ECRG4* (red curve), *ITIH5* (blue curve) and *ECRG4*-*ITIH5* (green curve) biomarker performance based on MSRE qPCR in cohort #2. (**C**) Scatter plot showed significant increased methylation values for *NID2* in urine sediments of bladder cancer compared to benign lesions, inflammatory and healthy samples; *** *p* < 0.001. (**D**) ROC-curve analysis compares *ECRG4* (red curve), ITIH5 (blue curve), and *NID2* (orange curve) and combined ECRG4-NID2 biomarker performance based on MSRE qPCR in cohort #2. *AUC*: Area under the curve.

Next, the biomarker quality of *ECRG4* and *ITIH5* promoter methylation was tested and then compared to a known putative bladder cancer methylation biomarker (*NID2*) by Methylation Sensitive Restriction Enzyme (MSRE) qPCR at the independent laboratories of Biotype GmbH (Dresden, Germany). The independent urine cohort (overall *n* = 211) included 130 urines of patients that were diagnosed with primary bladder cancer as well as 81 control urines (benign lesions, inflammatory diseases, and healthy controls). *ECRG4*-*ITIH5* methylation was found to be significantly increased in the urines of bladder cancer patients (mean methylation: 10.31, 95% CI: 6.132–14.50) as compared to all control groups, i.e., benign lesions (mean methylation: 0.143), inflammatory diseases (mean methylation: 2.900), and healthy donors (mean methylation: 0.203) (Figure 4A). In this independent cohort, a close association of both *ECRG4* and *ITIH5* with increased tumor size (pTa vs. pT1-4) and age at diagnosis was determined by Fisher’s exact test (Table 2 and Table 3). 

The ROC analyses showed that *ECRG4-ITIH5* combination achieved a sensitivity of 71.6% at a specificity of 80.2% (AUC: 0.771, 95% CI: 0.706–0.836) in a cohort that included benign lesions and inflammatory diseases of the urinary tract (Figure 4B), whereas specificity was increased up to 92% with minimally decreased sensitivity (69%) focusing on healthy controls. When comparing our single biomarkers, i.e., *ITIH5* and *ECRG4*, with the recently proposed biomarker candidate *NID2* [31], we revealed a similar biomarker performances of *ECRG4* and *NID2*, achieving a sensitivity of 73.1% (AUC: 0.780, 95% CI: 0.709–0.851) and 75% (AUC: 0.801, 95% CI: 0.729–0.873) at a specificity of 83.1%, respectively (Table 4). 

According to that, *ITIH5* was characterized by reduced sensitivity (56.7%, AUC: 0.674, 95% CI: 0.592–0.755), which did not lead to improved biomarker performance when combining with ECRG4. While considering that, we were able to increase sensitivity for bladder cancer detection up to 75% with improved specificity (85.9%; AUC: 0.807, 95% CI: 0.737–0.877) in a cohort comprising benign lesions and inflammatory diseases of the urinary tract when combining *ECRG4* with the known biomarker *NID2* (Figure 4C,D). In comparison to healthy controls, the panel set of *ECRG-NID2* achieved 76% sensitivity with 97.3% specificity (AUC: 0.884, 95% CI: 0.831–0.937) (Table 4).

## 3. Discussion

The field of liquid biopsy-based cancer detection systems is rapidly evolving, as novel (epi)genetic biomarkers have been characterized, which can be detected in biological fluids, like blood or urine, offering an easy and non-invasive application for cancer detection, prognosis, and therapy prediction. In colorectal cancer (CRC), for instance, a blood-based screening was realized by targeting Septin 9 (*SEPT9*) hypermethylation, whose Epi proColon® test has been approved by the FDA in 2016 [32]. In bladder cancer, liquid biopsy still needs improvement [33], as various molecular (epi)genetic biomarker candidates and signatures have been described, but none of those assays are FDA-approved for routine diagnostic so far. 

In the current study, we present two novel biomarker candidates, *ITIH5* and *ECRG4*, which show strong potential for improving or even completing existing non-invasive biomarker panels and platforms. Already in the year 2010, Renard and colleagues identified *TWIST1* and *NID2* as putative biomarker candidates while using qMSP [31]. In the same year, Costa et. al. showed three novel gene loci, i.e., *GDF15*, *TMEFF2*, and *VIM*, whose DNA methylation could be suitable for detecting bladder cancers in urine samples [34]. Meanwhile, many more putative candidates have been presented [16], but most of the studies were characterized by small sample cohorts without taking into account crucial cohorts of non-malignant diseases like chronic inflammation. Hence, only a handful of biomarkers such as *TMEFF2*, *NID2* and *TWIST1* meet to some degree the needed requirements and, thus, were independently studied and validated [35]. Therefore, we took great care to implement suitable steps and criteria for biomarker validation from the beginning of the study. In the first step, established biomarker assays were assessed by comparing the reliability of different detection methods (qMSP and pyrosequencing) in dependency on the urine sample processing *in vitro*. Of importance, we demonstrated that the regular procedure of urine processing analogue to SOPs of the centralized biomaterial bank (cBMB) of the RWTH Aachen University (i.e., storage at −80 °C) did not impair biomarker detection. However, the DNA yield was considerably higher when fresh urine samples were processed. Beyond that, the qMSP technique showed both the highest sensitivity and reproducibility, which is in line with previous studies demonstrating high levels of accuracy and lower rates of false negatives as compared with other techniques [36]. In a second step, we validated the clinical performance of both biomarkers by independent cohorts and laboratories. In this setting, we included high numbers of urological benign and inflammatory diseases as controls that can be endemically and frequently found in larger population groups, thereby reflecting a much more real-world scenario. We achieved a sensitivity ranging between 64 to 72% at a specificity of over 80% by combined performance of the *ECRG4*-*ITIH5* DNA methylation biomarker panel. Importantly, we still detect approximately 50% of bladder tumors with a robust true-negative rate (>80% specificity) by including urological malignancies of other origins supporting a liquid biopsy application in the field of bladder cancer. However, a putative benefit of both biomarker candidates being further useful for future assays dealing with the non-invasive detection of other urothelial malignancies, like prostate or renal cell carcinomas, should not be excluded at this stage. Interestingly, in our independent training cohort from Dresden, *ECRG4* reached a sensitivity of over 73% as single biomarker. Hence, a suitable impact was suggested, in particular for *ECRG4*, for discriminating BPH or urocystitis from bladder tumors, which is comparable with proposed biomarker candidates, like *NID2* or *TWIST1* [33]. *CFTR, SAL3,* and *TWIST1* have been recently shown to be useful for monitoring bladder cancer in a real clinical scenario, as a sensitivity of 96% was achieved by pyrosequencing in combination with urine cytology—however with low specificity (40%) [35]. In our cohort, the ECRG4-ITIH5 biomarker performance also reached over 90% sensitivity at a specificity of 40%, however, we finally focused on the best panel according to their specificity: combining *ECRG4* and *NID2* led to an increased sensitivity (76%) at a specificity of 97% when compared to healthy controls, encouraging validation studies of this biomarker setting in the future.

In view of novel diagnostic platforms, *ECRG4* and *ITIH5* could also be part of NGS-based gene signatures, which are currently considered to be at the cutting edge of the technical development of future diagnostic applications. In 2017, the multiplex bisulfite NGS-based sequencing concept “UroMark” was described achieving 98% sensitivity and 97% specificity [37]. However, this NGS assay should be confirmed in comprehensive cohorts and the usability of a 150 CpG loci comprising biomarker assay for routinely and cost-effective diagnosis, in particular as a population-based screening tool, in a real-world scenario must be further considered. So far, real-world application of available urinary markers has not reduced any bladder cancer treatment costs, as predicted by decision-analytic economic models [2]. Still, biomarkers are missing, which serve as the basis for decision-making of risk stratification. According to that, DNA methylation of our markers, in particular *ITIH5,* might hold a prognostic impact, as both candidates have been characterized as putative tumor suppressor genes whose silencing could be triggered by DNA promoter hypermethylation [20]. In 2008, *ITIH5* was described to be epigenetically silenced in breast cancer [17] and five years later *ITIH5* DNA methylation has been identified as a putative blood-based biomarker for the early detection of breast cancer [38]. Since then functionally studies revealed, for instance, ITIH5 mediated suppression of breast [21,22] and pancreatic cancer metastases [23] *in vitro* and *in vivo*. Interestingly, in aggressive mammary cancer cells, ITIH5 triggered an epigenetic reprogramming which was associated with a demethylation of various promoter regions, including that of *DAPK1*, a tumor suppressor gene and putative blood-based biomarker in several tumor entities [21]. In bladder carcinogenesis, the downregulation of ITIH5 was also associated with worse prognosis while functionally high-grade bladder cancer cells showed reduced growth *in vitro* after ITIH5 overexpression [20]. Of clinical interest, ITIH5 protein expression was shown to predict tumor relapse of the clinical important subgroup of pT1 high-grade patients [20], of which 30% never displayed recurrence after transurethral resection of the bladder, while a further 30% died due to metastatic disease [39]. In the present study, we now confirmed a putative prognostic impact as *ITIH5* promoter hypermethylation was associated with poor patients’ outcome in the subgroup of advanced tumors (pT > 2) of the TCGA bladder cancer data set, while increased *ITIH5* methylation was further shown to correlate with a higher pT status in our second urine cohort. These findings may support our hypothesis that *ITIH5* could be a useful biomarker for risk stratification, helping to monitor patients for the recurrence and/or progression of bladder tumors.

In conclusion, we provide two novel DNA methylation biomarkers for non-invasive detection of bladder carcinomas. As *ITIH5* might keep prognostic information for bladder cancer risk stratification, while *ECRG4* showed a convincing diagnostic performance, in particular in combination with the known biomarker candidate *NID2*, both biomarkers, *ECRG4* and *ITIH5*, may be promising candidates to complete and improve current biomarker panels and platforms. For instance, the “Bladder EpiCheck^TM^” urine assay that combines 15 DNA methylation biomarkers leading to an overall sensitivity of 68.2% and a specificity of 80.0% [40] may benefit from our biomarker candidates to reduce the number of biomarkers while also improving the overall performance. Future studies should be conducted to clarify which of our biomarkers, is suitable for which clinical application, e.g. as a guidance tool for early detection, risk stratification, surveillance, and/or therapeutic management.

## 4. Materials and Methods

### 4.1. Cell Line

The bladder cancer cell line J82 was originally obtained from the American Type Culture Collection (ATCC, Manassas, VA, USA). The urothelial bladder cancer cell line RT112 was used for studies of the analytical performance, a gift from Dr. Alexander Buchner (LMU München, München, Germany). All of the cell lines successfully underwent an identity check (Multiplexion GmbH, Immenstadt, Germany) prior to the experiments. 

### 4.2. Urine Samples

In total, 474 urine samples were assessed in this study. The Departments of Urology of the University Hospitals of Aachen, Bonn, and Dresden provided the voided urine samples. The samples that were collected in Aachen were obtained from the RWTH centralized biomaterial bank (RWTH cBMB). The collection of tissue samples was performed within the framework of the Biobank of the Center for Integrated Oncology Köln Bonn. All of the patients gave written consent for asservation and analysis of their samples according to local Institutional Review Board (IRB)-approved protocols of the Medical Faculty of RWTH Aachen University (EK 206/09, 05 Jan 2010), the University of Bonn (EK 205/13, 16 Mar 2013), and the University of Dresden (EK 96032012, 15 Jul 2014). The urine samples derived from patients diagnosed with a primary bladder tumor (*n* = 246) were used to assess biomarker performance, while samples with a known second malignancy, such as prostate cancer, were excluded from this study. Urines from healthy donors (*n* = 49) and samples derived from patients with inflammatory (chronic cystitis), benign (benign prostate hyperplasia), and urological malignant diseases of other tissue origin (testicular tumors, prostate cancer, renal cell carcinoma) served as the controls (overall *n* = 179). For the characteristics of training cohort I (Aachen–Bonn) and training cohort II (Dresden) see Table 5. Unless otherwise stated, 10–20 mL of urines were centrifuged for 10 min. at 2000 × g, washed with PBS and sediments were stored at −80 °C.

### 4.3. DNA Extraction from Urines

The urine sediments of training cohort I (Aachen–Bonn) stored at −80 °C were subjected to DNA extraction by using the ZR Urine DNA Isolation Kit (ZR, Zymo Research, Freiburg, Germany), following the manufacturer’s instructions. DNA extraction from the urine sediments of training cohort II (Dresden) stored at −80 °C in RLT buffer was performed by using the QIAamp DNA Mini Kit (Qiagen, Hilden, Germany), according to the manufacturer’s instructions. The DNA yield (ng/mL urine) and purity (A_260_/A_280_) were determined by using the NanoDrop (Thermo Fisher Scientific, Waltham, MA, USA). Only extractions from urines with a minimal total amount of 100 ng genomic DNA and a ratio of ≥1.5 were finally used for qMSP, pyrosequencing, and MSRE qPCR analyses. 

### 4.4. DNA Bisulfite Conversion 

100 to 250 ng of the genomic DNA (training cohort I) were bisulfite-converted for 14 to 16 h by using the EZ DNA Methylation™ kit (Zymo Research) according to the manufacturer’s instructions. Bisulfite-converted DNA was eluted in 20 µL of TRIS-EDTA buffer. 

### 4.5. Bisulfite-Pyrosequencing 

The pyrosequencing of bisulfite-converted DNA was performed by using the PyroMark PCR Kit, the PyroMark96 ID device, and the PyroGoldSQA reagent Kit (Qiagen), as reported previously [20]. *ECRG4* and *ITIH5* pyrosequencing assays were designed by using the Pyromark Assay Design Software (Qiagen), and Supplementary Table S3 lists all of the primers. Primers and sequence of interest meet the following criteria: Based on TCGA data analyses, sequences of interest should cover promoter regions that a) are characterized by strong differences in mean DNA methylation between urothelial normal and bladder cancer samples and b) are located in important gene regulatory sequences, i.e., a statistically significant inverse correlation between *ECRG4/ITIH5* gene expression and the corresponding DNA methylation had to be observed. The EpiTect® PCR Control DNA Set (Qiagen) was used as the positive controls for unmethylated and methylated DNA in each run.

### 4.6. Quantitative Methylation-Specific PCR (qMSP)

Bisulfite-modified DNA was used as a template for fluorescence-based real-time PCR amplified in an iCycler iQ5 (Biorad, Munich, Germany), as previously described [37] with slight modifications: The designed primers and probes were specific for amplifying bisulfite-converted DNA for the genes of interest (*ECRG4* and *ITIH5*) (for cycle conditions, primer sequences, and annealing temperatures, see Supplementary Table S4). The reference gene *GAPDH* was used for internal normalization. Eight calibration dilutions of *in vitro* methylated human leukocyte DNA (0.1%, 1%, 5%, 10%, 20%, 30%, 50%, 100%) and unmethylated sequence (human leukocyte DNA from a healthy donor), as well as multiple water blanks were included in each run. The gene of interest was called methylated if the cycle threshold (Ct) of at least two of three qPCR replicates for each specimen had a value of less than 45 cycles. The amount of methylated DNA (percentage of methylated reference, PMR) at a specific locus was calculated by dividing the GENE/*GAPDH* ratio of a sample by the GENE/*GAPDH* ratio of SssI-treated human leukocyte DNA and multiplying by 100, as specified [37]. The primer binding sites of the qMSP assays were located in the same genomic promoter region as covered by pyrosequencing. The efficiencies of real time MSP were calculated according to the equation: E = 10^[−1/slope of calibration dilutions]^ [41] and the mean efficacy of ECRG4 and ITIH5 qMSP was 76.57% and 77.76%, respectively.

### 4.7. Methylation Sensitive Restriction Enzyme qPCR (MSRE) qPCR

Isolated genomic DNA (125 ng) was used for double restriction digest. Methylation-sensitive restriction enzymes AciI and HpaII (New England Biolabs, NEB, MA, USA) were selected based on their capacity to distinguish methylated from unmethylated DNA sequences. Two independent digestion reactions (test reaction and control) were prepared for each patient DNA. Restriction digest was performed within a total volume of 25 µL in CutSmart Buffer (NEB) for 1 h at 37 °C and followed by heat inactivation for 20 min. at 80 °C. The control samples were treated in the same way but without the addition of the enzymes, 50% glycerol was added instead. Finally, DNA digest was diluted with 1x TE buffer before MSRE qPCR. The designed primers and probes for MSRE qPCR are specific for amplifying unrestricted DNA for the genes of interest (ECRG4, ITIH5, and NID2). 

MSRE qPCRs were carried out while using the Roche LightCycler 480 II Real-Time PCR detection system. Mono color hydrolysis probe detection (FAM) was used. All of the samples were done in duplicate in 25 μL reactions containing 5 µL Reaction Mix B (Biotype GmbH, Dresden), 3 U Multi Taq 2 (Biotype), 1.5 µL primers and probes (5 µM each), nuclease-free water (Biotype), and 2 µL of digested DNA (2.5 ng/µL, test or control template). For *ECRG4* and *NID2,* the addition of Combinatorial Enhancer Solution (1× CES, [42]) was necessary due to the very high GC content of the amplified region. 

For cycle conditions, primer sequences and annealing temperatures, see Supplementary Table S5. Ct values were analyzed while using LightCycler 480 Software (Hoffmann-La Roche AG, Basel, Switzerland).

Undetected Ct values were normalized to 47 for calculations. The methylation level of the amplified region was calculated by using the following equation: percent methylation = 100 × 2^−ΔCt^, where ΔCt is the average Ct value from the test reaction minus the average Ct values from the control reaction. Methylation values exceeding 100% were set to 100%.

### 4.8. Analytical Assay Performances 

RT112 wildtype bladder cancer cells harboring a methylated *ECRG4* and *ITIH5* promoter were cultured for two weeks. After cell counting RT112 cells were spiked into pooled urine of healthy donors (*n* = 4). Serial dilutions (10 to 10.000) of RT112 cells were added to 20 mL pooled urine, respectively, which was subsequently processed at the RWTH cBMB laboratories according to its standard operating protocol. Afterwards, urine pellets were either directly used for DNA extraction (probe set A) or urine sediments were stored according to the RWTH cBMB conditions by using two-dimensional (2D) barcoded LVL tubes (LVL technologies, Crailsheim, Germany) at −80 °C for two weeks (probe set B). Pooled urines without any spiked RT112 cells served as the control for normalization and threshold calculation by defining methylation cut-offs. Next, DNA was bisulfite-treated, as mentioned earlier, and the *ECRG4* and *ITIH5* qMSP as well as pyrosequencing assays were performed for *ECRG4* and *ITIH5*, respectively. The gene of interest was called methylated if the PMR (qMSP) or mean percent of CpG methylation (pyrosequencing) stably exceed the background noise and certainly maintained this threshold (=cut-off).

### 4.9. TCGA BLCA Data Set

Infinium HumanMethylation450 BeadChip data (level 2) and RNASeqV2 data (level 3) of the tumor and normal tissue samples were obtained from the TCGA data portal [30] and analyzed, as previously described [43].

### 4.10. Statistical Data Acquisition

Two-sided p-values that were less than 0.05 were considered to be significant. The non-parametric Mann–Whitney U-test was applied in order to compare two groups, whereas, in the case of more than two groups, the Dunn’s multiple comparison test was used. Correlation analysis was performed by calculating a non-parametric *Spearman’s rank* correlation coefficient. Statistical associations between clinico-pathological parameters and DNA methylation of *ITIH5* and *ECRG4* were determined by Fisher’s exact test by using SPSS software version 25.0 (SPSS Inc., Chicago, IL, USA). Survival curves for overall survival (OS) were calculated using the Kaplan–Meier method with log-rank statistics. OS was measured from surgery until death and it was censored for patients alive without evidence of death at the last follow-up date. The receiver operating characteristics (ROC) curves and AUC values were calculated to assess the biomarker performance of *ECRG4* and *ITIH5* methylation similar to our previous report [43]. The ROC curves of combined biomarkers were based on the binary logistic regression model using the probability as test variable.

## Figures and Tables

**Table 1:** EI-BLA biomarker performance based on training cohort #1 as compared to different control groups.

*EI-BLA qMSP*					
Cut-Off	Specificity	Sensitivity	AUC	*p*-Value	Control Group
0.54	81.5%	64.3%	0.783	<0.001	1a
0.38	81.6%	50.9%	0.695	<0.001	1b

**Table 2:** Clinico-pathological parameters in relation to ITIH5 methylation in training cohort #2.

		*ITIH5* Methylation ^b^			
		*n* ^a^	Low	High	*p*-Value ^c^
Age at diagnosis					
	≤70 years	111	65	46	**0.001**
	>70 years	106	39	67	
	Gender				
	male	143	57	56	0.150
	female	42	22	20	
Histological tumor grade ^d^					
	low grade	19	4	15	0.174
	high grade	113	42	71	
Tumor stage ^d^					
	pTa	76	33	43	**0.024**
	pT1-pT4	54	13	41	

^a^ Urines of cohort #2; ^b^ cut-off level MSRE = 0.22 representing >90% specificity in ROC curve statistic; ^c^ Fisher’s exact test; ^d^ According to WHO 2004 classification; Significant *p*-values are marked in bold face.

**Table 3:** Clinico-pathological parameters in relation to ECRG4 methylation in training cohort #2.

		*ECRG4* Methylation ^b^			
		*n* ^a^	Low	High	*p*-Value ^c^
Age at diagnosis					
	<70 years	111	84	27	**<0.001**
	≥70 years	106	55	51	
	Gender				
	male	143	82	61	0.802
	female	42	25	17	
Histological tumor grade ^d^					
	low grade	19	10	9	0.645
	high grade	113	53	60	
Tumor stage^d^					
	pTa	76	45	31	**0.007**
	pT1-pT4	54	19	35	

^a^ Urines of cohort #2; ^b^ cut-off level MSRE = 0.52 representing >90% specificity in ROC curve statistic; ^c^ Fisher’s exact test; ^d^ According to WHO 2004 classification; Significant *p*-values are marked in bold face.

**Table 4:** Biomarker performances based on training cohort #2.

**EI-BLA MRSE qPCR**					
Cut-Off	Specificity	Sensitivity	AUC	*p*-Value	Control Group
0.52	80.2%	71.6%	0.771	<0.001	all
0.53	91.9%	69.2%	0.850	<0.001	healthy
**Single markers MSRE qPCR (control group “all”)**					
Cut-Off	Specificity	Sensitivity	AUC	*p*-Value	Marker
0.94	83.1%	73.1%	0.780	<0.001	*ECRG4*
0.77	83.1%	56.7%	0.674	<0.001	*ITIH5*
0.11	83.1%	75.0%	0.801	<0.001	*NID2*
**Combined ECRG4-NID2 MSRE qPCR**					
Cut-Off	Specificity	Sensitivity	AUC	*p*-Value	Control Group
0.49	85.9%	75.0%	0.807	<0.001	all
0.49	97.3%	76.0%	0.884	<0.001	healthy

**Table 5:** The clinico-pathological parameters of 474 patients whose urine samples were analyzed in this study.

	Categorization	*n*	% Analyzable
**Controls**		228	100%
Age (median 61.0; range: 23–82 years)			
	<61.0 years	72	31.6%
	≥61.0 years	80	35.1%
	na	76	33.3%
Gender			
	male	96	42.1%
	female	24	10.5%
	na	108	47.4%
Diagnosis			
	Healthy	49	21.5%
	Uro-stones	13	5.7%
	Inflammatory—Uro-cystitis	38	16.7%
		
	Inflammatory—other	8	3.5%
	Benign—BPH	23	10.1%
	Benign—other	17	7.5%
	PCa	48	21.1%
	GTR	5	2.2%
	RCC	27	11.8%
**BCa-Asscociated ^a^**		246	100%
Age (median 70; range: 27–89 years)			
	<70 years	119	48.4%
	≥70 years	127	51.6%
Gender			
	male	195	79.3%
	female	51	20.7%
Histological tumor grade ^b^			
	low grade	42	17.1%
	high grade	172	69.9%
	na	32	13.0%
Tumor stage ^b^			
	pTa	106	43.1%
	pTis	8	3.3%
	pT1	54	22.0%
	pT2	37	15.0%
	pT3	19	7.7%
	pT4	8	3.3%
	pTx	8	3.3%
	na	6	2.4%

^a^ Only urine samples of patients preoperatively diagnosed with primary, bladder cancer (BCa, without any other malignancy) were included; ^b^ According to WHO 2004 classification; BPH: prostate hyperplasia; PCa: prostate cancer; GRT: germline tumor; RCC: renal cell carcinoma; na: not available

## Supplementary Materials

Supplementary materials can be found at https://www.mdpi.com/1422-0067/21/3/1117/s1.

## References

[1] Ferlay J., Colombet M., Soerjomataram I., Mathers C., Parkin D.M., Piñeros M., Znaor A., Bray F. (2019). Estimating the global cancer incidence and mortality in 2018: GLOBOCAN sources and methods. Int. J. Cancer.

[2] Yeung C., Dinh T., Lee J. (2014). The health economics of bladder cancer: An updated review of the published literature. Pharmacoeconomics.

[3] Leal J., Luengo-Fernandez R., Sullivan R., Witjes J.A. (2016). Economic Burden of Bladder Cancer Across the European Union. Eur. Urol..

[4] Schlake A., Crispen P.L., Cap A.P., Atkinson T., Davenport D., Preston D.M. (2012). NMP-22, urinary cytology, and cystoscopy: A 1 year comparison study. Can. J. Urol..

[5] National Collaborating Centre for Cancer (UK) (2015). Bladder Cancer: Diagnosis and Management.

[6] Sarosdy M.F., Kahn P.R., Ziffer M.D., Love W.R., Barkin J., Abara E.O., Jansz K., Bridge J.A., Johansson S.L., Persons D.L. (2006). Use of a multitarget fluorescence in situ hybridization assay to diagnose bladder cancer in patients with hematuria. J. Urol..

[7] Lotan Y., Roehrborn C.G. (2003). Sensitivity and specificity of commonly available bladder tumor markers versus cytology: Results of a comprehensive literature review and meta-analyses. Urology.

[8] Bhat A., Ritch C.R. (2019). Urinary biomarkers in bladder cancer: Where do we stand?. Curr. Opin. Urol..

[9] Bubendorf L. (2011). Multiprobe fluorescence in situ hybridization (UroVysion) for the detection of urothelial carcinoma - FISHing for the right catch. Acta Cytol..

[10] Behrens T., Stenzl A., Brüning T. (2014). Factors influencing false-positive results for nuclear matrix protein 22. Eur. Urol..

[11] Wang Z., Que H., Suo C., Han Z., Tao J., Huang Z., Ju X., Tan R., Gu M. (2017). Evaluation of the NMP22 BladderChek test for detecting bladder cancer: A systematic review and meta-analysis. Oncotarget.

[12] Pichler R., Fritz J., Tulchiner G., Klinglmair G., Soleiman A., Horninger W., Klocker H., Heidegger I. (2018). Increased accuracy of a novel mRNA-based urine test for bladder cancer surveillance. BJU Int..

[13] Ecke T.H., Weiß S., Stephan C., Hallmann S., Arndt C., Barski D., Otto T., Gerullis H. (2018). UBC® Rapid Test-A Urinary Point-of-Care (POC) Assay for Diagnosis of Bladder Cancer with a focus on Non-Muscle Invasive High-Grade Tumors: Results of a Multicenter-Study. Int. J. Mol. Sci..

[14] Baylin S.B., Jones P.A. (2011). A decade of exploring the cancer epigenome-biological and translational implications. Nat. Rev. Cancer..

[15] Molnár B., Tóth K., Barták B.K., Tulassay Z. (2015). Plasma methylated septin 9: A colorectal cancer screening marker. Expert Rev. Mol. Diagn..

[16] Larsen L.K., Lind G.E., Guldberg P., Dahl C. (2019). DNA-Methylation-Based Detection of Urological Cancer in Urine: Overview of Biomarkers and Considerations on Biomarker Design, Source of DNA, and Detection Technologies. Int. J. Mol. Sci..

[17] Veeck J., Chorovicer M., Naami A., Breuer E., Zafrakas M., Bektas N., Dürst M., Kristiansen G., Wild P.J., Hartmann A. (2008). The extracellular matrix protein ITIH5 is a novel prognostic marker in invasive node-negative breast cancer and its aberrant expression is caused by promoter hypermethylation. Oncogene.

[18] Kloten V., Rose M., Kaspar S., von Stillfried S., Knüchel R., Dahl E. (2014). Epigenetic inactivation of the novel candidate tumor suppressor gene ITIH5 in colon cancer predicts unfavorable overall survival in the CpG island methylator phenotype. Epigenetics.

[19] Dötsch M.M., Kloten V., Schlensog M., Heide T., Braunschweig T., Veeck J., Petersen I., Knüchel R., Dahl E. (2015). Low expression of ITIH5 in adenocarcinoma of the lung is associated with unfavorable patients’ outcome. Epigenetics.

[20] Rose M., Gaisa N.T., Antony P., Fiedler D., Heidenreich A., Otto W., Denzinger S., Bertz S., Hartmann A., Karl A. (2014). Epigenetic inactivation of ITIH5 promotes bladder cancer progression and predicts early relapse of pT1 high-grade urothelial tumours. Carcinogenesis.

[21] Rose M., Kloten V., Noetzel E., Gola L., Ehling J., Heide T., Meurer S.K., Gaiko-Shcherbak A., Sechi A.S., Huth S. (2017). ITIH5 mediates epigenetic reprogramming of breast cancer cells. Mol. Cancer.

[22] Rose M., Meurer S.K., Kloten V., Weiskirchen R., Denecke B., Antonopoulos W., Deckert M., Knüchel R., Dahl E. (2018). ITIH5 induces a shift in TGF-*β* superfamily signaling involving Endoglin and reduces risk for breast cancer metastasis and tumor death. Mol. Carcinog..

[23] Sasaki K., Kurahara H., Young E.D., Natsugoe S., Ijichi A., Iwakuma T., Welch D.R. (2017). Genome-wide in vivo RNAi screen identifies ITIH5 as a metastasis suppressor in pancreatic cancer. Clin. Exp. Metastasis.

[24] Yue C.M., Deng D.J., Bi M.X., Guo L.P., Lu S.H. (2003). Expression of ECRG4, a novel esophageal cancer-related gene, downregulated by CpG island hypermethylation in human esophageal squamous cell carcinoma. World J. Gastroenterol..

[25] Li L.W., Yu X.Y., Yang Y., Zhang C.P., Guo L.P., Lu S.H. (2009). Expression of esophageal cancer related gene 4 (ECRG4), a novel tumor suppressor gene, in esophageal cancer and its inhibitory effect on the tumor growth in vitro and in vivo. Int. J. Cancer.

[26] Tang G.Y., Tang G.J., Yin L., Chao C., Zhou R., Ren G.P., Chen J.Y., Zhang W. (2019). ECRG4 acts as a tumor suppressor gene frequently hypermethylated in human breast cancer. Biosci. Rep..

[27] Luo L., Wu J., Xie J., Xia L., Qian X., Cai Z., Li Z. (2016). Downregulated ECRG4 is associated with poor prognosis in renal cell cancer and is regulated by promoter DNA methylation. Tumour Biol..

[28] Götze S., Feldhaus V., Traska T., Wolter M., Reifenberger G., Tannapfel A., Kuhnen C., Martin D., Müller O., Sievers S. (2009). ECRG4 is a candidate tumor suppressor gene frequently hypermethylated in colorectal carcinoma and glioma. BMC Cancer.

[29] Cai Z., Liang P., Xuan J., Wan J., Guo H. (2016). ECRG4 as a novel tumor suppressor gene inhibits colorectal cancer cell growth in vitro and in vivo. Tumour Biol..

[30] Cancer Genome Atlas Research Network (2014). Comprehensive molecular characterization of urothelial bladder carcinoma. Nature.

[31] Renard I., Joniau S., van Cleynenbreugel B., Collette C., Naômé C., Vlassenbroeck I., Nicolas H., de Leval J., Straub J., Van Criekinge W. (2010). Identification and validation of the methylated TWIST1 and NID2 genes through real-time methylation-specific polymerase chain reaction assays for the noninvasive detection of primary bladder cancer in urine samples. Eur. Urol..

[32] Song L., Jia J., Peng X., Xiao W., Li Y. (2017). The performance of the SEPT9 gene methylation assay and a comparison with other CRC screening tests: A meta-analysis. Sci. Rep..

[33] Lodewijk I., Dueñas M., Rubio C., Munera-Maravilla E., Segovia C., Bernardini A., Teijeira A., Paramio J.M., Suárez-Cabrera C. (2018). Liquid Biopsy Biomarkers in Bladder Cancer: A Current Need for Patient Diagnosis and Monitoring. Int. J. Mol. Sci..

[34] Costa V.L., Henrique R., Danielsen S.A., Duarte-Pereira S., Eknaes M., Skotheim R.I., Rodrigues A., Magalhães J.S., Oliveira J., Lothe R.A. (2010). Three epigenetic biomarkers, GDF15, TMEFF2, and VIM, accurately predict bladder cancer from DNA-based analyses of urine samples. Clin. Cancer Res..

[35] Van der Heijden A.G., Mengual L., Ingelmo-Torres M., Lozano J.J., van Rijt-van de Westerlo C.C.M., Baixauli M., Geavlete B., Moldoveanud C., Ene C., Dinney C.P. (2018). Urine cell-based DNA methylation classifier for monitoring bladder cancer. Clin. Epigenetics.

[36] Hernández H.G., Tse M.Y., Pang S.C., Arboleda H., Forero D.A. (2013). Optimizing methodologies for PCR-based DNA methylation analysis. Biotechniques.

[37] Feber A., Dhami P., Dong L., de Winter P., Tan W.S., Martínez-Fernández M., Paul D.S., Hynes-Allen A., Rezaee S., Gurung P. (2017). UroMark-a urinary biomarker assay for the detection of bladder cancer. Clin. Epigenetics.

[38] Kloten V., Becker B., Winner K., Schrauder M.G., Fasching P.A., Anzeneder T., Veeck J., Hartmann A., Knüchel R., Dahl E. (2013). Promoter hypermethylation of the tumor-suppressor genes ITIH5, DKK3, and RASSF1A as novel biomarkers for blood-based breast cancer screening. Breast Cancer Res..

[39] Shahin O., Thalmann G.N., Rentsch C., Mazzucchelli L., Studer U.E. (2003). A retrospective analysis of 153 patients treated with or without intravesical bacillus Calmette-Guerin for primary stage T1 grade 3 bladder cancer: Recurrence, progression and survival. J. Urol..

[40] Witjes J.A., Morote J., Cornel E.B., Gakis G., van Valenberg F.J.P., Lozano F., Sternberg I.A., Willemsen E., Hegemann M.L., Paitan Y. (2018). Performance of the Bladder EpiCheck™ Methylation Test for Patients Under Surveillance for Non-muscle-invasive Bladder Cancer: Results of a Multicenter, Prospective, Blinded Clinical Trial. Eur. Urol. Oncol..

[41] Pfaffl M.W. (2001). A new mathematical model for relative quantification in real-time RT-PCR. Nucleic Acids Res..

[42] Ralser M., Querfurth R., Warnatz H.J., Lehrach H., Yaspo M.L., Krobitsch S. (2006). An efficient and economic enhancer mix for PCR. Biochem. Biophys. Res. Commun..

[43] Mijnes J., Veeck J., Gaisa N.T., Burghardt E., de Ruijter T.C., Gostek S., Dahl E., Pfister D., Schmid S.C., Knüchel R. (2018). Promoter methylation of DNA damage repair (DDR) genes in human tumor entities: RBBP8/CtIP is almost exclusively methylated in bladder cancer. Clin. Epigenetics.

