# Supplementary material for: ITIH5 and ECRG4 DNA Methylation Biomarker Test (EI-BLA) for Urine-Based Non-Invasive Detection of Bladder Cancer
Source: Int J Mol Sci. 2020 Feb 7;21(3):1117. doi: 10.3390/ijms21031117 (PMC7036997; doi:10.3390/ijms21031117)
Supplement: Supplementary file 1 [file ijms-21-01117-s001.zip › ijms-683106-supplementary/IJMS-683106Supplemental Figure S1-S2.docx]

**Supplemental Figures**


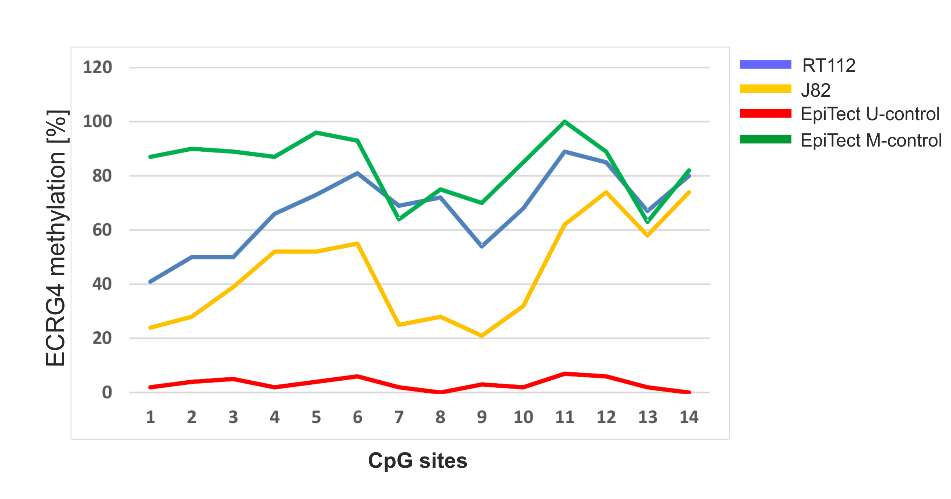


Supplementary Figure S1: *ECRG4* promoter methylation of 14 CpG sites determined by pyrosequencing of RT112 (blue line)and J82 (orange line) bladder cancer cells. The EpiTect® PCR Control DNA Set (Qiagen) was used as positive controls for unmethylated (red line) and completely methylated DNA (green line).


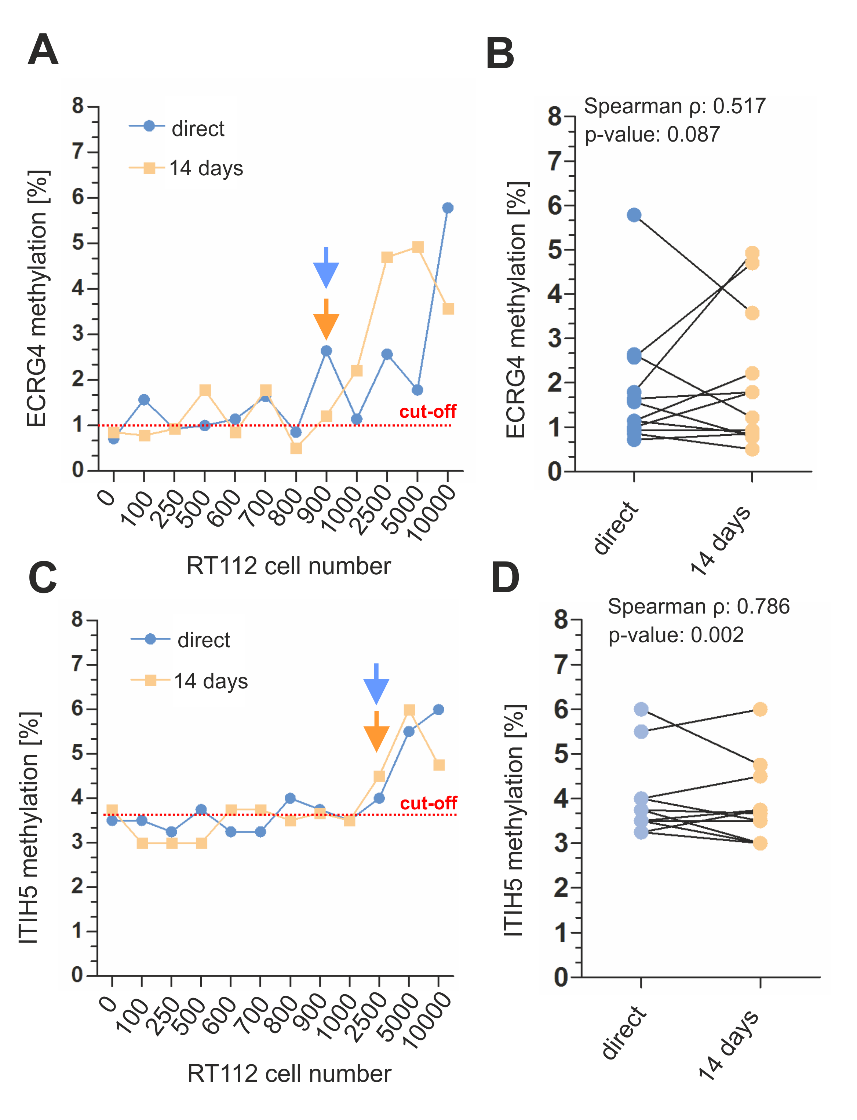


Supplementary Figure S2: (**A)** *ECRG4* promoter methylation determined by using pyrosequencing of spiked urines samples. Red dotted line: threshold value for positive detection; orange and blue arrow: stably exceeding the threshold (detection limit) **(B)** Correlation of ECRG4 DNA methylation for spiked urine samples of probe set A and B. (**C)** *ITIH5* promoter methylation determined by using pyrosequencing of spiked urines samples. Red dotted line: threshold value for positive detection; orange and blue arrow: stably exceeding the threshold (detection limit) **(D)** Correlation of ITIH5 DNA methylation for spiked urine samples of probe set A and B.
