# Supplementary material for: ITIH5 and ECRG4 DNA Methylation Biomarker Test (EI-BLA) for Urine-Based Non-Invasive Detection of Bladder Cancer
Source: Int J Mol Sci. 2020 Feb 7;21(3):1117. doi: 10.3390/ijms21031117 (PMC7036997; doi:10.3390/ijms21031117)
Supplement: Supplementary file 1 [file ijms-21-01117-s001.zip › ijms-683106-supplementary/ijms-683106supplementary Table S1-S5..docx]

**Supplemental Tables**

| **Supplementary Table S1:** | | | | | |  | |  |
| --- | --- | --- | --- | --- | --- | --- | --- | --- |
| **Clinico-pathological parameters in relation to ITIH5 methylation in trainings cohort #1** | | | | | | | |  |
|  |  |  |  |  |  |  |  |  |
|  | | | ***ITIH5* methylation^b^** | | | | |  |
|  | | | ***n****^a^* | **low** | **high** | **P-value**^c^ | |  |
|  | | | | | |  | |  |
| Age at diagnosis | | |  |  |  |  | |  |
|  | ≤66 years | | 94 | 74 | 20 | 0.369 | |  |
|  | >66 years | | 89 | 65 | 24 |  |  |  |
| Gender | | | | | |  | |  |
|  | male | | 150 | 115 | 35 | 0.633 | |  |
|  | female | | 33 | 24 | 9 |  |  |  |
| Histological tumor grade^d^ | | |  |  |  |  | |  |
|  | low grade | | 23 | 14 | 9 | 0.920 | |  |
|  | high grade | | 57 | 34 | 23 |  |  |  |
| Tumor stage^d^ | | |  |  |  |  | |  |
|  | pTa | | 63 | 42 | 20 | 0.508 | |  |
|  | pT1-pT4 | | 36 | 22 | 14 |  |  |  |
| ^a^Urines of cohort #1; ^b^cut-off level PMR=0.35 representig >90% specificity in ROC curve statistic; ^c^Fisher’s exact test; ^d^According to WHO 2004 classification; Significant P-values are marked in bold face.   \| **Supplementary Table S2:** \| \| \| \| \|  \| \| --- \| --- \| --- \| --- \| --- \| --- \| \| **Clinico-pathological parameters in relation to ECRG4 methylation in trainings cohort #1** \| \| \| \| \| \| \| \|  \| \| ***ECRG4* methylation^b^** \| \| \| \| \|  \| \| ***n****^a^* \| **low** \| **high** \| **P-value**^c^ \| \|  \| \| \| \| \|  \| \| Age at diagnosis \| \|  \|  \|  \|  \| \|  \| ≤66 years \| 94 \| 71 \| 23 \| 0.969 \| \|  \| >66 years \| 89 \| 67 \| 22 \| \| Gender \| \| \| \| \|  \| \|  \| male \| 150 \| 117 \| 33 \| 0.084 \| \|  \| female \| 33 \| 21 \| 12 \| \| Histological tumor grade^d^ \| \|  \|  \|  \|  \| \|  \| low grade \| 23 \| 15 \| 8 \| 0.548 \| \|  \| high grade \| 57 \| 33 \| 24 \| \| Tumor stage^d^ \| \|  \|  \|  \|  \| \|  \| pTa \| 62 \| 37 \| 25 \| 0.889 \| \|  \| pT1-pT4 \| 36 \| 22 \| 14 \| \| ^a^Urines of cohort #1; ^b^cut-off level PMR=0.43 representig >90% specificity in ROC curve statistic; ^c^Fisher’s exact test; ^d^According to WHO 2004 classification; Significant P-values are marked in bold face. \| \| \| \| \| \| \| \| | | | | | | | |  |
|  |  |  |  |  |  |  |  |  |
|  |  |  |  |  |  |  |  |  |
| **Supplementary Table S3:** | | | | | | | | |
| **Primer sequences and PCR conditions for pyrosequencing analyses** | | | | | | | | |
|  | |  | | | | |  | |
| **Primer** | | **Sequence** | | | | | **Anealing temp [°C]** | |
|  |  |  |  |  |  |  |  |  |
| **ITIH5 PCR For** | | 5’-Biotin-TTYGGGAGGTAGTGGGTGTTGGTAAGAA-3’ | | | | | 56 | |
| **ITIH5 PCR Rev** | | 5’-CTCTATTCRAAAAAAAAACATTCCAAACCTAC-3’ | | | | |  |  |
| **ITIH5 sequencing** | | 5’-AAAAAAAAACATTCCAAAC-3’ | | | | |  | |
| **ECRG4 PCR For** | | 5’-GGGGAGGGAGAGAGGATTT-3’ | | | | | 56 | |
| **ECRG4 PCR Rev** | | 5’-Biotin-ACCCCATCAAAACCAAAACAACAAAC-3’ | | | | |  |  |
| **ECRG4 sequencing** | | 5’-GGGAGGGAGAGAGGATTT-3’ | | | | |  | |
|  | |  | | | | |  | |
| **pre-PCR cycle conditions:** 95°C for 5 min, 40 cycles of 95°C for 20 s, 56°C for 20 s, 72°C for 20 s and a final extension at 72°C for 10 min. | | | | | | | | |
|  |  |  |  |  |  |  |  |  |

| **Supplementary Table S4:** | | |
| --- | --- | --- |
| **Primer sequences and PCR conditions for qMSP analyses** | | |
|  |  |  |
| **Primer** | **Sequence** | **Anealing temp [°C]** |
|  |  |  |
| **qMSP ITIH5 For** | 5’-CAACACAAATAACCCCTACTATACG-3’ | 62 |
| **qMSP ITIH5 Rev** | 5’-TTTTCGGTTTTAGTTTTATTAGAGTCG-3’ |  |
| **qMSP ITIH5 probe** | 5’-6-Fam-ACGAAACAAACTTAAAATACCTTCTCCCCG-BHQ-1-3’ |  |
| **qMSP ECRG4 For** | 5’-GAGAGAGGATTTCGGTGGTATTCG-3’ | 66 |
| **qMSP ECRG4 Rev** | 5’-GAATTATCCCTACGTCGCTACCGA-3’ |  |
| **qMSP ECRG4 probe** | 5’-6-Fam-ACGAAACCCAACGAATAAACGCCGCG-BHQ-1-3’ |  |
| **qMSP GAPDH For** | 5’-GAGGATATAGTTTGGTTT TG-3’ | 58 |
| **qMSP GAPDH Rev** | 5’-CCTACCTAATAATAATCTTTACTT A-3’ |  |
| **qMSP GAPDH probe** | 5’-6-Fam-ACTCCAATCCCTAACCCTACCTTT-BHQ-1-3’ |  |
|  |  |  |
| **qMSP cycle conditions:** | | |
| Amplifications were carried out by using the following profile: 95°C for 10 minutes, 50 cycles at 95°C for 15 seconds, and primer annealing at 58°C to 66°C for 30 seconds. | | |
|  |  |  |
|  |  |  |

| **Supplementary Table S5:** | | |
| --- | --- | --- |
| **Primer sequences and PCR conditions for MSRE qPCR analyses** | | |
|  |  |  |
| **Primer** | **Sequence** | **Anealing temp [°C]** |
|  |  |  |
| **qPCR ITIH5 For** | 5’-CGCAAAGAAGCATATTGCAC-3’ | 60 |
| **qPCR ITIH5 Rev** | 5’-TAGCTCTGTTCGGGGAGAAG-3’ |  |
| **qPCR ITIH5 Probe** | 5’-6-Fam-CCCCTCCCACACCCTCCCAT-3’ |  |
| **qPCR ECRG4 For** | 5’-GAGAGAGGACCTCGGTGGTACT-3’ | 60 |
| **qPCR ECRG4 Rev** | 5’-CGCGGCCGCGGGTTATC-3’ |  |
| **qPCR ECRG4 Probe** | 5’-6-Fam-CCGCTTGGCCCTCAGCCCTCTGGC-3’ |  |
| **qPCR NID2 For** | 5’-TCCCCCTCCATGCTCGCTC-3’ | 60 |
| **qPCR NID2 Rev** | 5’-TAGCTTGCTGGGTGGGCCTG-3’ |  |
| **qPCR NID2 Probe** | 5’-6-Fam-CCTCCAGCCCACTCTCCGCGCCGC-3’ |  |
|  |  |  |
| qPCR cycle conditions: 94°C for 5 min, 47 cycles of 96°C for 30 s, 60°C for 30 s, 72°C for 60 s. | | |
|  |  |  |
